# EphB4 mediates resistance to antiangiogenic therapy in experimental glioma

**DOI:** 10.1007/s10456-018-9633-6

**Published:** 2018-07-10

**Authors:** Christian Uhl, Moritz Markel, Thomas Broggini, Melina Nieminen, Irina Kremenetskaia, Peter Vajkoczy, Marcus Czabanka

**Affiliations:** Department of Neurosurgery, Universitätsmedizin Charite – Campus Mitte, Luisenstrasse 46, 10117 Berlin, Germany

**Keywords:** Glioblastoma multiforme, Ephrin, EphB4, Antiangiogenic therapy, Pericyte, Intravital microscopy

## Abstract

**Introduction:**

Alterations in vascular morphogenesis are hallmarks of antiangiogenesis-resistant tumor vessels. Vascular morphogenesis is regulated by ephrinB2-EphB4 system which may induce different biological effects depending on the oncological and molecular contexts. It was the aim of the current study to characterize the influence of EphB4 on tumor microcirculation after antiangiogenic treatment using different SF126 glioma models.

**Materials and methods:**

Using an ecotropic transfection system, empty vector (pLXSN) or EphB4 (EphB4^OE^) overexpressing Phoenix-ECO cells were coimplanted with SF126 glioma cells subcutaneously (dorsal skinfold chamber, DSC) and orthotopically (cranial window, CW). Tumor volume was assessed by MRI. Intravital microscopy (IVM) allowed microcirculatory analysis (total {TVD} and functional vessel density {FVD}, diameter {D}, and permeability index {PI}) before and after antiangiogenic treatment (Sunitinib: DSC: 40 mg/kg BW, 6 days; CW: 80 mg/kg BW, 4 days). Immunohistochemistry included Pecam–Desmin, Ki67, TUNEL, and Caspase 3 stainings.

**Results:**

EphB4^OE^ induced large and treatment-resistant tumor vessels (*FVD: Control*/*Su: 110* ± *23 cm*/*cm*^*2*^ *vs. EphB4*^*OE*^/*Su: 103* ± *42 cm*/*cm*^*2*^). Maintenance of pericyte–endothelial cell interactions (*Control: 80* ± *12 vs. Control*/*Su: 47* ± *26%; EphB4*^*OE*^: *88* ± *9 vs. EphB4*^*OE*^/*Su: 74* ± *25%*) and reduced antiproliferative (*Control: 637* ± *80 vs. Control*/*Su: 110* ± *22; EphB4*^*OE*^: *298* ± *108 vs. EphB4*^*OE*^/*Su: 213* ± *80*) and proapoptotic responses (*Control: 196* ± *25 vs. Control* / *Su: 404* ± *60; EphB4*^*OE*^: *183* ± *20 vs. EphB4*^*OE*^/*Su: 270* ± *66*) were observed under EphB4 overexpression.

**Conclusion:**

EphB4 overexpression leads to vascular resistance by altering vascular morphogenesis, pericyte coverage, and cellular proliferation/apoptosis in experimental SF126 glioma models.

## Introduction

In clinical human glioblastoma treatment, antiangiogenic therapy has failed to meet the initially high expectations with different clinical trials failing to demonstrate a clinical benefit for different antiangiogenic agents [1]. Despite the initial description of antiangiogenesis as a strategy resistant to resistance [2], these trials demonstrate that resistance mechanisms play a major role in clinical glioma treatment [1]. Adaptive and intrinsic resistance mechanisms have been described in this regard [3]. Especially, increased pericyte coverage has been proposed as a contributor to resistance [3, 4]. Recent data demonstrate that pericyte-independent mechanisms significantly contribute to resistance against antiangiogenic therapy underlining the role of altered vascular morphogenesis as a major characteristic of therapy-resistant glioma microvessels. The clinical significance of EphB4 signaling in human glioma has been emphasized recently identifying the ephrinB2-EphB4 system as a clinically relevant molecular pathway governing progression-free survival and glioma growth in patients [5]. The ephrinB2-EphB4 system represents one of the most important regulators of vascular morphogenesis in glioma angiogenesis by mediating pericyte–endothelial cell interactions [5, 6]. Interestingly sunitinib-resistant and temozolomide-resistant glioma vessels demonstrate the same vascular phenotype as EphB4 overexpressing glioma vessels [4, 5, 7]. However, different biological consequences of ephrinB2-EphB4 signaling have been described depending on the oncological and biological contexts [8]. Therefore, it was the aim of the current study to investigate the influence of EphB4 overexpression on microcirculation and vascular resistance using SF126 glioma cells in different experimental approaches.

## Materials and methods

### Study design

EphB4-overexpressing or control (pLXSN) Phoenix-ECO reporter cells were coimplanted with SF126 glioma cells subcutaneously and orthotopically into nude mice. After reaching a tumor volume of 50 mm^3^ in the subcutaneous xenograft model, low-dose antiangiogenic treatment with VEGF-receptor 2 blocker sunitinib (40 mg/kg BW i.p.) was initiated for 7 days. Placebo groups received injections of 0.9% NaCl.

Tumor volume in the orthotopic xenografts was measured by small-animal MRI on days 21, 25, and 29 postoperatively (p.o.). Treatment with high-dose sunitinib (80 mg/kg BW i.p.) or placebo was initiated for 4 days, starting on day 25 p.o. After growth analysis, xenografts were harvested, perfused with PBS and PFA, and stained immunohistochemically to determine pericyte coverage, proliferating, and apoptotic cell nuclei. For each model, four experimental groups emerged: control (pLXSN), treated control (pLXSN + Sunitinib), EphB4 overexpression (EphB4^OE^), and treated EphB4-overexpression (EphB4^OE^ + Sunitinib).

For detailed microvascular analysis, EphB4^OE^- and pLXSN-reporter cells were coimplanted with SF126 glioma cells into dorsal skinfold chambers and as 3D-spheroids into chronic cranial windows to determine total vessel density (TVD), functional vessel density (FVD), vessel diameter, and volumetrical blood flow. Intravital microscopy (IVM) of the tumors in the dorsal skinfold chamber was performed on days 11, 14, and 18 p.o. Low-dose antiangiogenic treatment with sunitinib or a placebo was administered between days 12 and 17 p.o. In the chronic cranial window, IVM was performed on days 12, 14, and 16 p.o. High-dose antiangiogenic treatment with sunitinib was applied for 4 days starting on day 12 p.o.

## Mice

Athymic NU/NU Nude Mice (Charles River Laboratories, Sulzfeld, Germany) were operated on when they reached an age of 6–8 weeks. All animals were kept according to the National Institutes of Health guide for the care and use of Laboratory animals. Experiments were approved by the responsible state institution (Landesamt für Gesundheit und Soziales, Berlin) (TVA—G0144/13).

## Cell lines

SF126 cells were purchased from JCRB Cell Bank (Order ID: F1500012, Cell number: IFO50286). SF126 glioma cells and Phoenix-ECO cells (A.Ullrich/U.Eichelsbacher, Martinsried, Germany) were utilized, which either contained an empty pLXSN vector for control or pLXSN vectors that had undergone cloning in of the entire coding region of the EphB4 receptor (2992 bp). Creation of retroviral systems and their vectors has been described by Millauer et al. [9]. Cell lines were cultivated with 4.5 g/l in Dulbecco’s Modified Eagle’s Medium (Gibco™, Karlsruhe, Germany), which was supplied with 10% fetal bovine serum (ScienCell, San Diego, USA) and 1% penicillin–streptomycin solution (ScienCell, San Diego, USA) at 37 °C at 5% CO_2_. Geneticin disulfate (Roth, Karlsruhe, Germany) 800 µg/ml was added to select Phoenix-ECO cells for neomycin resistance.

### Protein extraction and western blotting

Cell lysates were prepared by incubation in RIPA lysis buffer with protease inhibitor cocktail (Thermo Scientific, #1860932). Quantification of total protein concentration was performed with BCA protein assay (Pierce, #23227). The samples were boiled in LDS loading buffer containing 5% β-mercaptoethanol. 20 µg protein/well were loaded on a 6% SDS resolving gel. The samples were run in Mini-PROTEAN® Tetra cell electrophoresis system (Bio-Rad) at 150 V for 60 min. Proteins were then transferred to 0.45 µm (for EphB4) PVDF membrane (Millipore, #IPFL 000 10) using Bio-Rad mini TransBlot cell system with plate at 0.4 A for 2 h. Tris-buffered saline (TBS) with 0.05% Tween20 (TBST) was used for all wash steps. Membranes were blocked in the Starting Block T20 (TBS) blocking buffer (Thermo Scientific, #37543) for 1 h at room temperature, followed by an overnight incubation with the primary antibody (goat anti-mouse EphB4, R&D Systems, #AF446, dilution 1:500) at 4 °C. HRP-conjugated donkey anti-goat antibody was used as a secondary antibody (Jackson ImmunoResearch, #705-035-147, dilution 1:10,000). For loading control, anti-actin-HRP antibody (Sigma-Aldrich, #A3854, dilution 1:10,000) was used. The membranes were incubated with anti-actin-HRP antibody for 60 min at room temperature. ECL reaction was performed using SuperSignal Femto Substrate (Thermo Scientific #34095).

### Ecotropic retrovirus transfection system

**Fig. 1:**
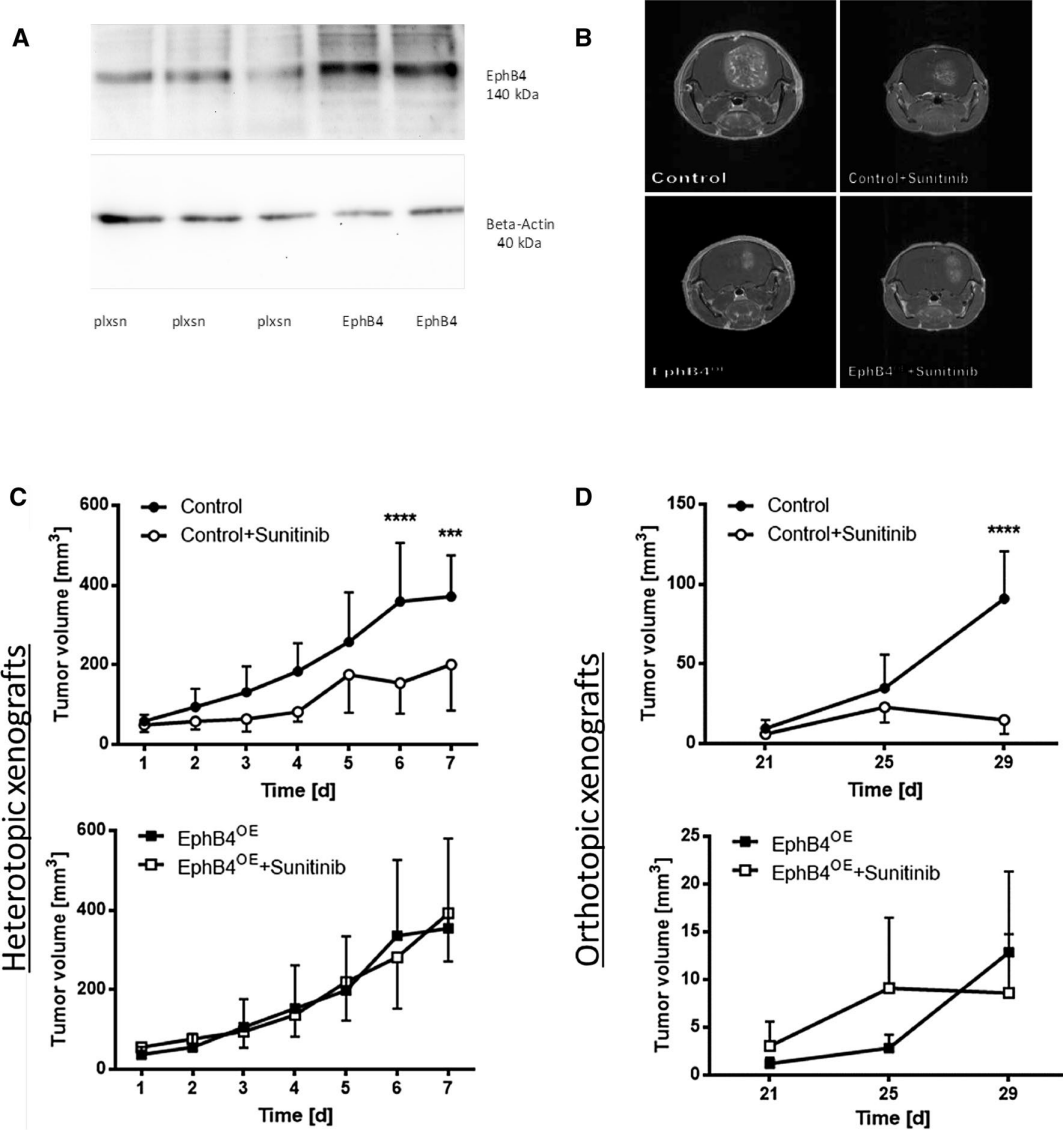
EphB4 overexpression results in therapy resistance against sunitinib treatment. **a** Western blot analysis of EphB4 in orthotopic xenografts following coimplantation of SF126 glioma cells and EphB4-overexpressing or control (pLXSN) Phoenix-ECO cells respectively. **b** Sample pictures of MRI scans of orthotopic xenografts on day 29 p.o. Untreated control tumors presented large tumors, while treated control tumors displayed significantly smaller tumor loads; no visible difference between treated and untreated EphB4^OE^-tumors. **c** Growth curves of the heterotopic xenografts. No reduction in tumor growth was observed in treated EphB4^OE^-tumors. ***p < 0.001, ****p < 0.0001; all groups *n* = 8. All values are displayed as mean ± SD. **d** Growth curves of orthotopic xenografts. No reduction in tumor growth was observed in treated EphB4^OE^-tumors. ****p < 0.0001; Control *n* = 6, Control + Sunitinib *n* = 6, EphB4^OE^ *n* = 6, EphB4^OE^ + Sunitnib *n* = 7. All values are displayed as mean ± SD

To selectively investigate the influence of endothelial EphB4 expression on vascular resistance mechanisms, Erber et al. designed and generated a rodent-specific ecotropic retroviral vector (pLXSN) containing cDNA encoding full-length EphB4 (EphB4) [10]. Using these constructs, stable virus-producing clones of Phoenix E producer cells were generated. In our human xenograft model (SF 126 glioma), a rodent-specific ecotropic retrovirus selectively infects mouse endothelial cells, leading to an exclusive expression of the transgene in the tumor vascular system [10]. Erber et al. demonstrated, in various experiments, high selectivity of this system for surface expression of EphB4 in transfected cell clones, rodent specificity of ecotropic viruses, no infection of human SF126 glioma cells by the used retrovirus, EphB4 overexpression in transfected cell clones, and increased EphB4 tyrosine phosphorylation compared to control viruses [10]. As this system has been established and intensively described previously, we did not repeat the basic establishing experiments as they are consuming time and resources. To verify successful application of the ecotropic retrovirus transfection system, we analyzed EphB4 expression in tumors as shown in Fig. 1.

### Subcutaneous xenografts

2 × 10^6^ Phoenix-ECO and SF126 glioma cells, respectively, were merged in PBS and coimplanted *via* subcutaneous injection into the flanks of nude mice. All groups *n* = *8*.

### Orthotopic xenografts

1 × 10^4^ Phoenix-ECO and SF126 glioma cells, respectively, were merged in PBS and coimplanted *via* stereotactic implantation into nude mice. PLXSN *n* = *6*, pLXSN + Sun *n* = *6*, EphB4^OE^ *n* = *6*, and EphB4^OE^ + Sun *n* = *7*.

### Dorsal skinfold chamber

2 × 10^6^ Phoenix-ECO and SF126 glioma cells, respectively, were merged in PBS and implanted in the dorsal skinfold chamber. Procedure of the operation has been described before [11]. All groups *n* = *6*.

### Chronic cranial window

3D-spheroid essay, as described by Laib et al., was performed with 2.5 × 10^4^ Phoenix-ECO and SF126 glioma cells respectively. Chronic cranial window operation was performed, as described by Foltz et al. [6]. All groups *n* = *5*.

### Intravital microscopy

IVM as well as determination of TVD, FVD, and vessel diameter was performed as described before [5, 11]. TVD was defined as the length of all vessels per ROI (0.06 cm × 0.05 cm) [cm/cm^2^]. Using a computer-based microcirculation analysis software (CapImage™), length of all vessels (cm) was measured in relation to the area of the analyzed ROI(cm^2^). This included all vessels perfused by FITC-Dextran as well as vessels that were non-functional and thereby did not contain any fluorescence dye or demonstrated motionless blood flow (static erythrocytes). FVD was defined as the length of all vessels that were perfused by FITC-Dextran or displayed moving erythrocytes per ROI [cm/cm^2^]. For the assessment of microvascular diameter, a diagonal line was drawn from the left upper corner to the right lower corner of the intended ROI and diameter of all vessels that were cut by the diagonal were analyzed to guarantee randomized evaluation and observer-independent analysis. The diameter was determined as the length of one vessel wall to another [µm].

### Immunohistochemistry

Orthotopic and subcutaneous xenografts were counterstained for CD31 (BD Biosciences, Heidelberg, Germany) and Desmin (Abcam, Cambridge, UK) to determine pericyte coverage [%] as described by Erber et al. [10]. Proliferating cell nuclei were assessed by Ki67-staining (Thermo Scientific, Fremont, California) and apoptotic cell nuclei by means of caspase-3 staining (Sigma–Aldrich, Munich, Germany) in the dorsal skinfold chamber and TUNEL staining in the chronic cranial window respectively (ApopTag® Red-In-Situ-Apoptosis-Detection-Kit, Merck Millipore, Darmstadt, Germany). Of each tumor, 3 slices were evaluated; of each slice, 5 pictures in ×20 in the respective ROI (1.93 mm × 1.51 mm) were taken. Desmin–Pecam ratio was assessed manually by counting desmin positive and negative vessels per ROI and by calculating the ratio between them (desmin positive vessels/desmin positive and negative vessels; %). Evaluation of apoptotic cell nuclei and Ki67-positive cells was performed by means of automatic computation using Image J with an installed Cell Counter as plugin. The green fluorescent channel for Ki67 and the rhodamine channel for TUNEL staining or Caspase staining, respectively, were singled out, converted to 8-bit format and, subsequently, converted to binary images to display all Ki67-positive cells and all TUNEL-positive or Caspase-positive cell nuclei, respectively, as black pixels on a white background. These pixels were counted automatically by the cell counter to determine the proliferating cells and apoptotic cell nuclei for each region of interest [*n*/ROI].

### Statistical analysis

Data were evaluated using GraphPad Prism 5 (GraphPad Software, San Diego, USA). Comparison of the different groups in the dorsal skinfold chamber, the chronic cranial window, the subcutaneous implantations, and the orthotopic xenografts was performed using two-way ANOVA with Sidak correction with the threshold for significance being *p* ≤ 0.05 (as in all other experiments). The differences in pericyte coverage, proliferating, and apoptotic cell nuclei in the immunohistological stainings were evaluated using one-way ANOVA with Bonferroni correction. Results are presented as mean ± standard deviation.

## Results

### EphB4 overexpression mediates tumor resistance to sunitinib treatment

Coimplantation of SF126 and Phoenix-ECO cells resulted in successful overexpression of EphB4 in endothelial cells (Fig. 1a). In both experimental glioma models, control tumors treated with sunitinib displayed a significantly reduced tumor volume compared to untreated controls (Fig. 1b–d). In EphB4 overexpression groups, tumor volume was not affected by sunitinib treatment displaying similar growth behavior between placebo- and sunitinib-treated groups both in the subcutaneous and orthotopic model (Fig. 1b–d).

**Fig. 2:**
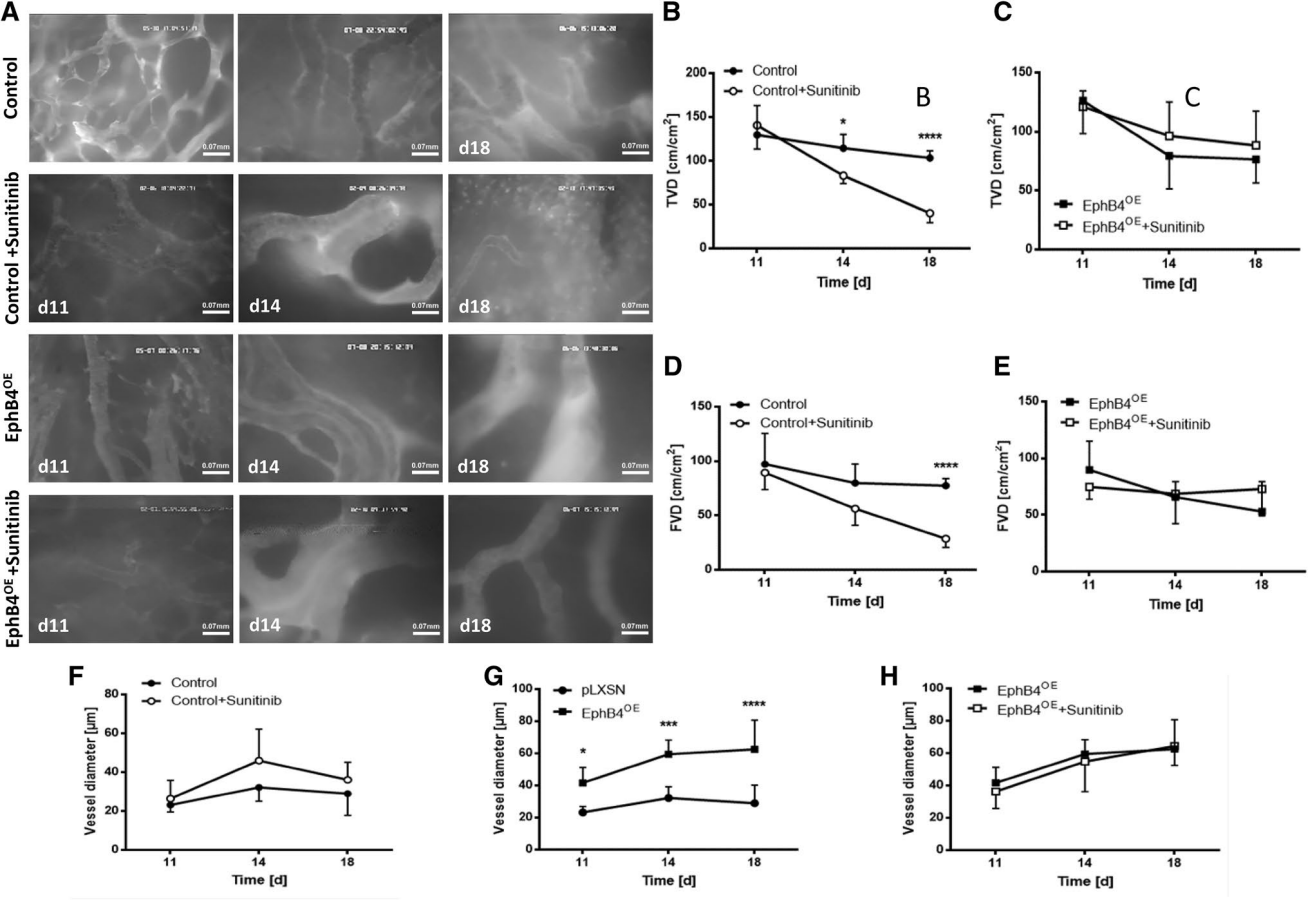
Therapy-resistant tumor vessels demonstrate significant morphological differences compared to control vessels in the dorsal skinfold chamber. **a** Sample pictures of IVM in the dorsal skinfold chamber on the respective postoperative days demonstrating selection of large tumor vessels under EphB4 overexpression similar to therapy-resistant vessels in treated controls; these altered tumor vessels do not respond to antiangiogenic treatment. Scale bars as indicated. **b**–**e** Evaluation of TVD and FVD: a significant reduction of TVD (*p < 0.05, ****p < 0.0001) on day 14 and 18 p.o. and FVD (****p < 0.0001) on day 18 p.o. in control tumors; all groups *n* = 6. All values are displayed as mean ± SD. **f**–**h** No difference in vessel diameter neither among control nor among EphB4^OE^-tumors following treatment. EphB4^OE^ vessels were significantly larger than the ones of the control group. *p < 0.05, ***p < 0.001, ****p < 0.0001; all groups *n* = 6. All values are displayed as mean ± SD

**Fig. 3:**
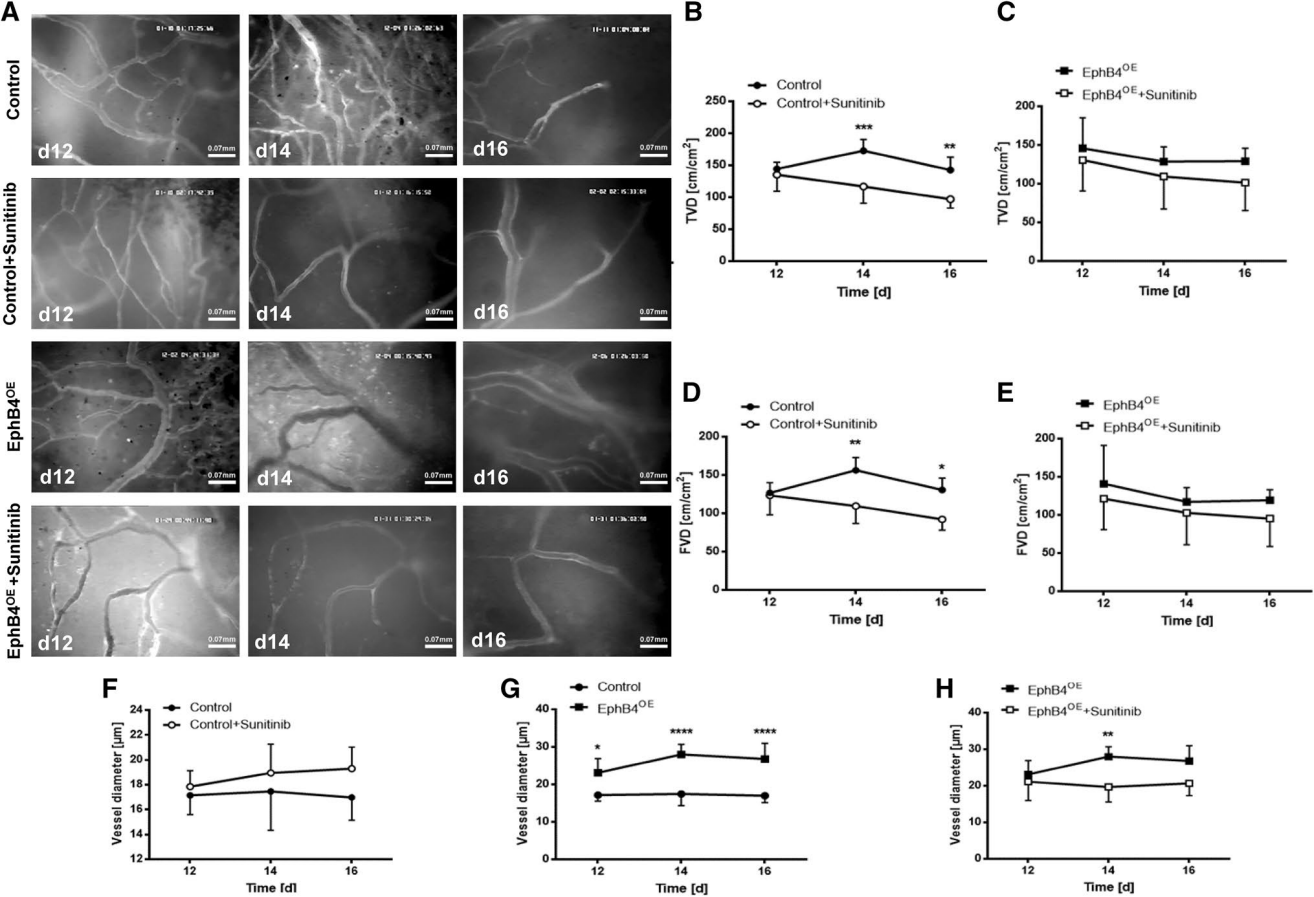
EphB4^OE^ tumors revealed sustained functioning vessel networks following antiangiogenic treatment in the chronic cranial window. **a** Sample pictures of IVM in the chronic cranial window on the respective postoperative days, demonstrating also increased diameter in resistant tumor vessels and in EphB4 overexpression glioma vessels; EphB4 overexpression vessels do not respond to sunitinib treatment. Scale bars as indicated. **b**–**e** Evaluation of TVD and FVD: a significant reduction of TVD (**p < 0.01, ***p < 0.001; all groups *n* = 5) and FVD (*p < 0.5, **p < 0.01; all groups *n* = 5) in treated control tumors on day 14 and 16 p.o. All values are presented as mean ± SD. **f**–**h** Vessel enlarging effect observed in therapy-resistant control vessels as well as in EphB4^OE^-vessels (*p < 0.05, **p < 0.001, ****p < 0.0001; all groups *n* = 5); EphB4 overexpressing glioma vessels do not respond with further vascular enlargement to sunitinib treatment while demonstrating resistance towards antiangiogenic treatment. All values are displayed as mean ± SD

Intravital microscopy demonstrated significantly reduced FVD and TVD in response to sunitinib treatment in controls while, in EphB4 overexpressing groups, sunitinib treatment did not reduce either TVD or FVD both in both experimental settings (Figs. 2, 3). Furthermore, altered vascular morphology was observed in treatment-resistant tumor vessels. EphB4 overexpression led to a significant increase in vessel diameter compared to control tumors (Figs. 2g, 3g). These vessels did not respond to antiangiogenic intervention with sunitinib as mentioned above. In control tumors, sunitinib treatment induced a trend towards increased vessel diameter, whereas, in treated EphB4 overexpressing tumors, vessel diameter was unchanged compared to placebo treatment (Figs. 2f–h, 3f–h).

### Pericyte–endothelial cell interactions and cellular proliferation are altered by antiangiogenic treatment

**Fig. 4:**
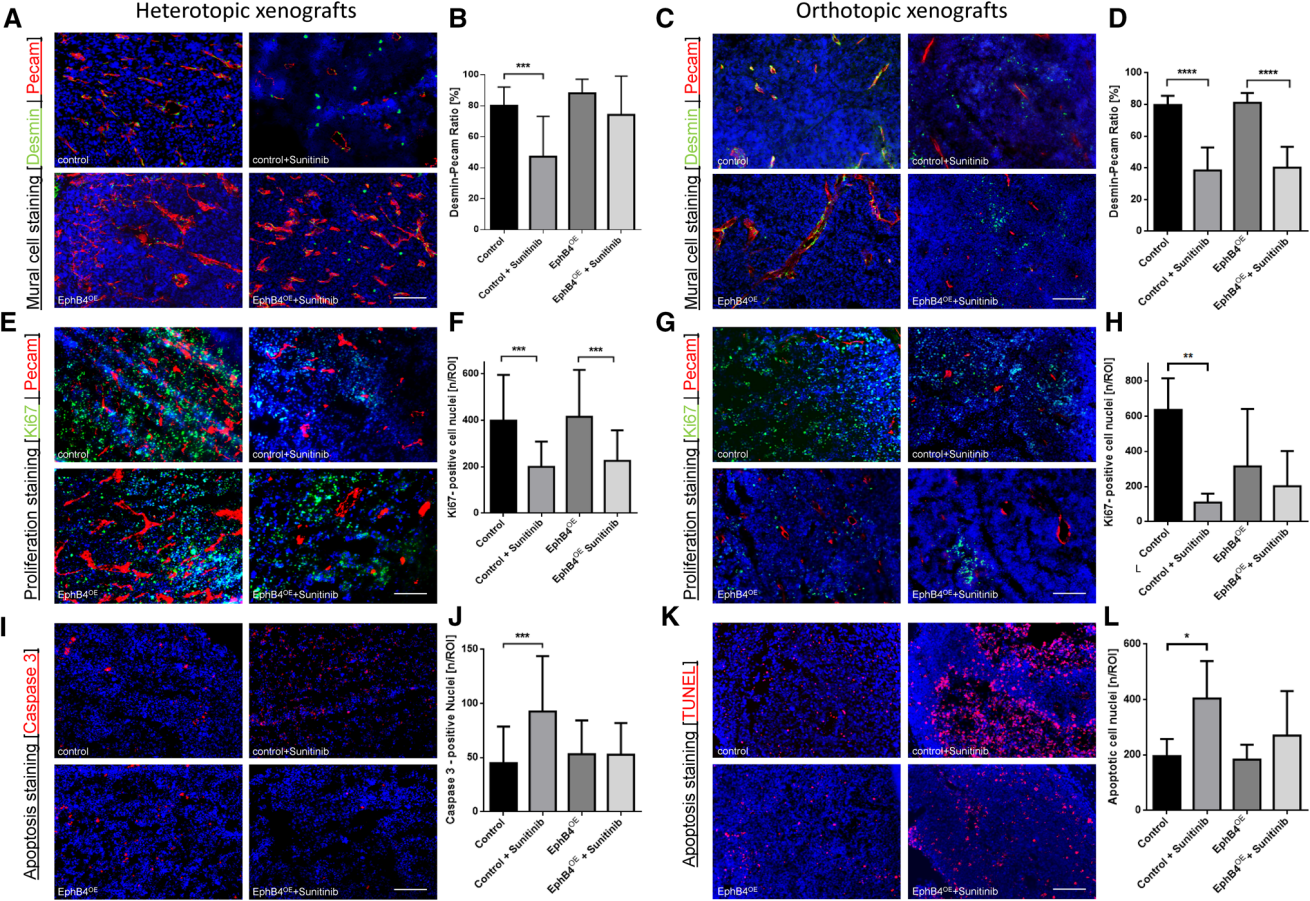
Effects of antiangiogenic treatment on pericyte–endothelial cell interaction, tumor cell proliferation, and tumor cell apoptosis. **a** Immunohistochemical double staining of pericytes and endothelial cells in the subcutaneous xenografts. Scale bar indicates 50 µm. **b** Quantification of desmin–pecam ratio in heterotopic xenografts. EphB4^OE^ tumors maintained extensive coverage of endothelial cells with pericytes during treatment. ***p < 0.001; all groups *n* = 5. All values are displayed as mean ± SD. **c** Immunohistochemical double staining of pericytes and endothelial cells in orthotopic xenografts. Scale bar indicates 50 µm. **d** Quantification of desmin–pecam ratio in the orthotopic xenografts. Ratio was significantly reduced in both treated groups. ****p < 0.0001; Control: *n* = 8, Control + Sunitinib: *n* = 5, EphB4^OE^: *n* = 5, EphB4^OE^ + Sunitinib: *n* = 5. All values are displayed as mean ± SD. **e** Sample images of Ki67 staining in the subcutaneous xenografts. Scale bar indicates 50 µm. **f** Quantification of Ki67-positive cell nuclei in subcutaneous xenografts. A significant reduction of proliferating cell nuclei in both treated groups. ***p < 0.001; all groups *n* = 4. All values are displayed as mean ± SD. **g** Sample images of Ki67-pecam double staining in orthotopic xenografts. Scale bar indicates 50 µm. **h** Quantification of Ki67-positive cell nuclei in the orthotopic xenografts. EphB4^OE^ tumors revealed significantly reduced proliferating cell nuclei, but, in contrast to control tumors, treatment did not reduce cellular proliferation. **p < 0.01; Control: *n* = 5, Control + Sunitinib: *n* = 5, EphB4^OE^: *n* = 7, EphB4^OE^ + Sunitinib: *n* = 6. All values are displayed as mean ± SD. **i** Sample images of Caspase 3 staining in the subcutaneous xenografts. Scale bar indicates 50 µm. **j** Quantification of apoptotic cell nuclei. A significant increase of apoptotic cell nuclei in treated control tumors compared to placebo-treated controls. ***p < 0.01; all groups *n* = 4. All values are displayed as mean ± SD. **k** Sample images of TUNEL staining in the orthotopic xenografts. Scale bar indicates 50 µm. **l** Evaluation of apoptotic cell nuclei. A significant increase of apoptotic cell nuclei in treated control tumors. *p < 0.05, Control: *n* = 6, Control + Sunitinib: *n* = 5, EphB4^OE^: *n* = 7, EphB4^OE^ + Sunitinib: *n* = 6. All values are displayed as mean ± SD

Sunitinib treatment led to a significant reduction of pericyte–endothelial cell interactions in control tumors (Fig. 4a–d). In subcutaneous xenografts, endothelial EphB4 overexpression stabilized and maintained pericyte–endothelial cell interactions without a significant difference between placebo- and sunitinib-treated groups (Fig. 4a, b). In the orthotopic glioma model, maintenance of pericyte–endothelial cell interactions was not observed for sunitinib treatment under EphB4 overexpression indicating pericyte-independent resistance mechanisms (Fig. 4c, d).

Cellular proliferation was assessed by Ki67 staining demonstrating significantly reduced proliferation in sunitinib-treated controls compared to placebo treatment in both experimental models (Fig. 4e–h). In the subcutaneous model, EphB4 overexpressing groups also showed a significant reduction of proliferation in response to sunitinib treatment compared to placebo (Fig. 4e, f). In orthotopically implanted glioma, reduction of cellular proliferation was not induced by sunitinib treatment, (Fig. 4g, h). No difference between sunitinib-treated control tumors and placebo-treated EphB4 overexpressing tumors was observed.

### EphB4 overexpression inhibits sunitinib-induced apoptosis

EphB4 overexpression did not alter rate of apoptosis between control and EphB4 overexpression groups in both experimental models (Fig. 4i–l). In control groups, sunitinib treatment induced a significant increase in apoptosis rate. This phenomenon was not observed under EphB4 overexpression in both experimental strategies, showing that endothelial EphB4 overexpression protects against the proapoptotic effects of sunitinib.

## Discussion

Endothelial EphB4 overexpression induces resistance against antiangiogenic therapy in SF126 glioma by altering vascular morphogenesis shifting the microvascular environment towards large, therapy-resistant microvessels. Alterations in microvascular blood flow as well as changes in cellular proliferation, apoptosis rate, and pericyte-endothelial cell interactions represent important mechanisms which are involved in this process.

Selection of therapy-resistant tumor vessels represents a key feature of evasive resistance mechanisms against antiangiogenic therapy [3]. Therapy-resistant vasculature in glioma has been characterized by distinct changes in morphogenesis. Combined temozolomide and sunitinib treatment leads to selection of large, high-flow, therapy-resistant glioma vessels by aggravating pericyte-mediated resistance mechanisms *via* angiopoetin–Tie 2 and delta-like-4 (Dll4)/notch signaling [4]. Dll4 expressing gliomas have been shown to be resistant towards anti-VEGF therapy by activating different angiogenesis-related pathways leading to large, resistant microvessels [7]. Pericyte–endothelial cell interactions are important regulators of vascular morphogenesis and represent key players in vascular resistance mechanisms [3]. Endothelial EphB4 overexpression has been shown to regulate glioma microvascular morphogenesis by aggravating pericyte–endothelial cell interactions [10]. We demonstrate that endothelial EphB4 overexpression leads to maintenance of pericyte–endothelial interactions despite effective anti-VEGF and anti-PDGF treatment leading to resistant glioma vasculature in a subcutaneous SF126 glioma model. In the orthotopic microenvironment, EphB4 overexpression induced significant alterations of vascular morphogenesis with a significant increase in microvascular diameter without altering the number of pericyte–endothelial cell interactions. In this regard, Erber et al. demonstrated that EphB4 overexpression leads to a change in the quality of pericyte–endothelial cell interactions with a tight pericyte–endothelial cell interface as compared to loose pericyte–endothelial cell interactions in controls without altering the number of pericyte–endothelial cell interactions [12].

Reduced pericyte–endothelial cell interactions may be the result of the high sunitinib dose used in the orthotopic experiments. The dose was applied purposely as this approach was used in the previous experiments to induce a relevant antiglioma effect in orthrotopically implanted experimental glioma [12]. Despite reduction of pericyte–endothelial cell interactions, therapy resistance was maintained. Similar findings were described by Li et al., showing that Dll4 expressing gliomas are characterized by large, bevacizumab-resistant microvessels despite significantly reduced pericyte–endothelial cell interactions [7]. The authors demonstrated, in detail, that inhibition of EphB4 abolished vascular resistance to bevacizumab [7]. One hypothesis explaining pericyte-independent vascular resistance mechanisms focuses on large and high-flow tumor vessels (as a result of EphB4 overexpression) providing superior supply of oxygen and nutrients which in turn leads to reduced expression of HIF1α and diminished VEGF dependence of the tumor with reduced endothelial expression of VEGFR [13]. Observations of improved microvascular hemodynamics were described previously with significantly increased microvascular delivery of chemotherapy in sunitinib-resistant glioma vasculature [13]. Other potential pericyte-independent mechanisms may include activation of other angiogenesis-related molecules that maintain angiogenesis signaling despite anti-VEGF therapy [3]. Apart from vascular resistance mechanisms, endothelial EphB4 overexpression induced a reduction of proapoptotic and antiproliferative effects of sunitinib indicating resistance mechanisms in glioma cells. Interactions between endothelial cells and glioma cells define the perivascular niche in glioma development [12]. In this regard, EphB4 overexpression reduced glioma growth in orthotopically implanted glioma in our study by leading to reduced proliferation. These observations are opposed by studies from Chen et al. demonstrating increased glioma growth in response to EphB4 upregulation mediated by increased epidermal growth factor receptor activity (EGFR) [14]. In human glioblastoma multiforme patients, high EphB4 expression correlates with decreased progression-free survival underlining a more aggressive phenotype [15]. However, ephrinB2-EphB4-mediated (onco)biological effects are extremely variable depending on tumor biology, microenvironment, presence or absence of ligand-dependent and ligand-independent signaling, in addition to forward and backward signaling mechanisms. In contrast to the above-named studies, which investigated the effects of EphB4 expression in tumor cells, we analyzed the effects of endothelial EphB4 overexpression. Moreover, discrepancies may further be explained by different ligand-independent and ligand-dependent effects of EphB4. In this regard, EphB4 has been shown to exert tumor progressive effects in the case of EphB4 overproduction in a ligand-independent mechanism, while ligand-dependent stimulation acts tumor suppressive [16]. Recent experiments depicted reverse signaling, mediated *via* ephrinB2, to act tumor promoting despite destabilizing tumor vascularization following EphB4 overexpression in malignant melanoma [17]. In human glioblastoma multiforme, a large heterogeneity exists in the expression of EphB4 and ephrinB2 depending on the biological characteristics of glioma cells and the associated microenvironment [18]. This heterogeneity is additionally complicated by the current changes in neuropathological classification of brain tumors [19]. It becomes clear that brain tumors are classified beyond the WHO classification system based on a molecular fingerprint (e.g., methylation status) which explains the very variable pathological and clinical courses observed in clinical treatment of the disease [19]. Therefore, it may be speculated that different expression profiles of ephrinB2 and EphB4 and the associated equilibrium between receptor and ligand account for the reported different effects of EphB4 overexpression in glioma biology. To address these issues, we used one glioma cell line and analyzed the effects of endothelial EphB4 overexpression on vascular resistance using defined hetero- and orthotopic experimental models. Further studies will have to focus on EphB4 mediated effects using different glioma cell lines and loss of function approaches to characterize EphB4 signaling depending on the oncobiological context to support clinical translation of EphB4 as a potential target in glioma resistance.
